# Advances in metabolome information retrieval: turning chemistry into biology. Part I: analytical chemistry of the metabolome

**DOI:** 10.1007/s10545-017-0074-y

**Published:** 2017-08-24

**Authors:** Abdellah Tebani, Carlos Afonso, Soumeya Bekri

**Affiliations:** 1 Department of Metabolic Biochemistry, Rouen University Hospital, 76000 Rouen, France; 2 Normandie Université, UNIROUEN, CHU Rouen, IRIB, INSERM U1245, 76000 Rouen, France; 3 Normandie Université, UNIROUEN, INSA Rouen, CNRS, COBRA, 76000 Rouen, France

## Abstract

Metabolites are small molecules produced by enzymatic reactions in a given organism. Metabolomics or metabolic phenotyping is a well-established omics aimed at comprehensively assessing metabolites in biological systems. These comprehensive analyses use analytical platforms, mainly nuclear magnetic resonance spectroscopy and mass spectrometry, along with associated separation methods to gather qualitative and quantitative data. Metabolomics holistically evaluates biological systems in an unbiased, data-driven approach that may ultimately support generation of hypotheses. The approach inherently allows the molecular characterization of a biological sample with regard to both internal (genetics) and environmental (exosome, microbiome) influences. Metabolomics workflows are based on whether the investigator knows a priori what kind of metabolites to assess. Thus, a targeted metabolomics approach is defined as a quantitative analysis (absolute concentrations are determined) or a semiquantitative analysis (relative intensities are determined) of a set of metabolites that are possibly linked to common chemical classes or a selected metabolic pathway. An untargeted metabolomics approach is a semiquantitative analysis of the largest possible number of metabolites contained in a biological sample. This is part I of a review intending to give an overview of the state of the art of major metabolic phenotyping technologies. Furthermore, their inherent analytical advantages and limits regarding experimental design, sample handling, standardization and workflow challenges are discussed.

## Introduction: A historical perspective

Systems biology is a new scientific paradigm aimed at unveiling the systemic function of biology and bridging the gap between biological information and its context. Systems biology can be defined as a global and systemic analysis of complex system interconnections and their functional interrelationships (Kitano 2002a, b; Kitano 2002a, b; Ehrenberg et al 2003; Weston and Hood 2004). Two seminal inputs have facilitated the emergence of systems biology: data generation and data modeling. High-throughput omics technologies allowed the recovery of a holistic and comprehensive biological information, but the development of computational capabilities have allowed sophisticated systems modeling and convenient visualization tools (Ritchie et al 2015; McMurry et al 2016; Tenenbaum et al 2016). Omics strategies aim at a comprehensive assessment of entire classes of biomolecules (genes, proteins, metabolites, etc.) of a biological tissue, cell, fluid, or organism. Conceptually, metabolomics has its roots in the practices of ancient Greek doctors who used the organoleptic characteristics of urine for diagnosis; for example, urine sweetness reveals the high glucose levels in diabetes. Such organoleptic chemical features are, of course, linked to metabolism. Olivier et al coined the metabolome in 1998 and defined it as the set of metabolites synthesized by an organism (Oliver et al 1998). Metabolome refers to all metabolites present in a given biological system, fluid, cell, or tissue (Nicholson et al 1999). Other terms have been used, including metabolic fingerprinting, metabolic footprinting, metabotyping, and metabolic phenotyping, with the latter being increasingly accepted. Metabolites can be defined as organic small molecules produced by enzymatic reactions. Thus, metabolomics is one of the “omic” technologies. It is based on biochemical and molecular characterizations of the metabolome and the changes in metabolites related to genetic, environmental, drug, or dietary variables in addition to other factors (Fiehn 2002; Holmes et al 2008; Dunn et al 2011a, 2011b; Benton et al 2012). Metabolomics has found different applications in many disease studies and in complex diseases, with promising perspectives in screening, diagnosis, prognosis, patient stratification, and treatment follow-up (Bekri 2016; Tebani et al 2016a, b). Metabolomics is the study of the complete biochemical profile, and the main analytical platforms are nuclear magnetic resonance (NMR) spectroscopy and mass spectrometry (MS) paired with separation methods such a high-performance liquid chromatography (HPLC). Metabolomics holistically investigates biological systems using an unbiased, data-driven approach that may ultimately lead to generation of hypotheses. In this review, major metabolic phenotyping technologies and their characteristics will be presented along with the challenges associated with data analysis. A discussion on current trends and the requirements for biomarker discovery will also be presented. Finally, we address the current state of the art with respect to standardization and workflow challenges and the gaps in preclinical and clinical environments that hinder translation of metabolic signatures into clinically useful tools.

## Analytical strategies and chemical information extraction

**Fig. 1:**
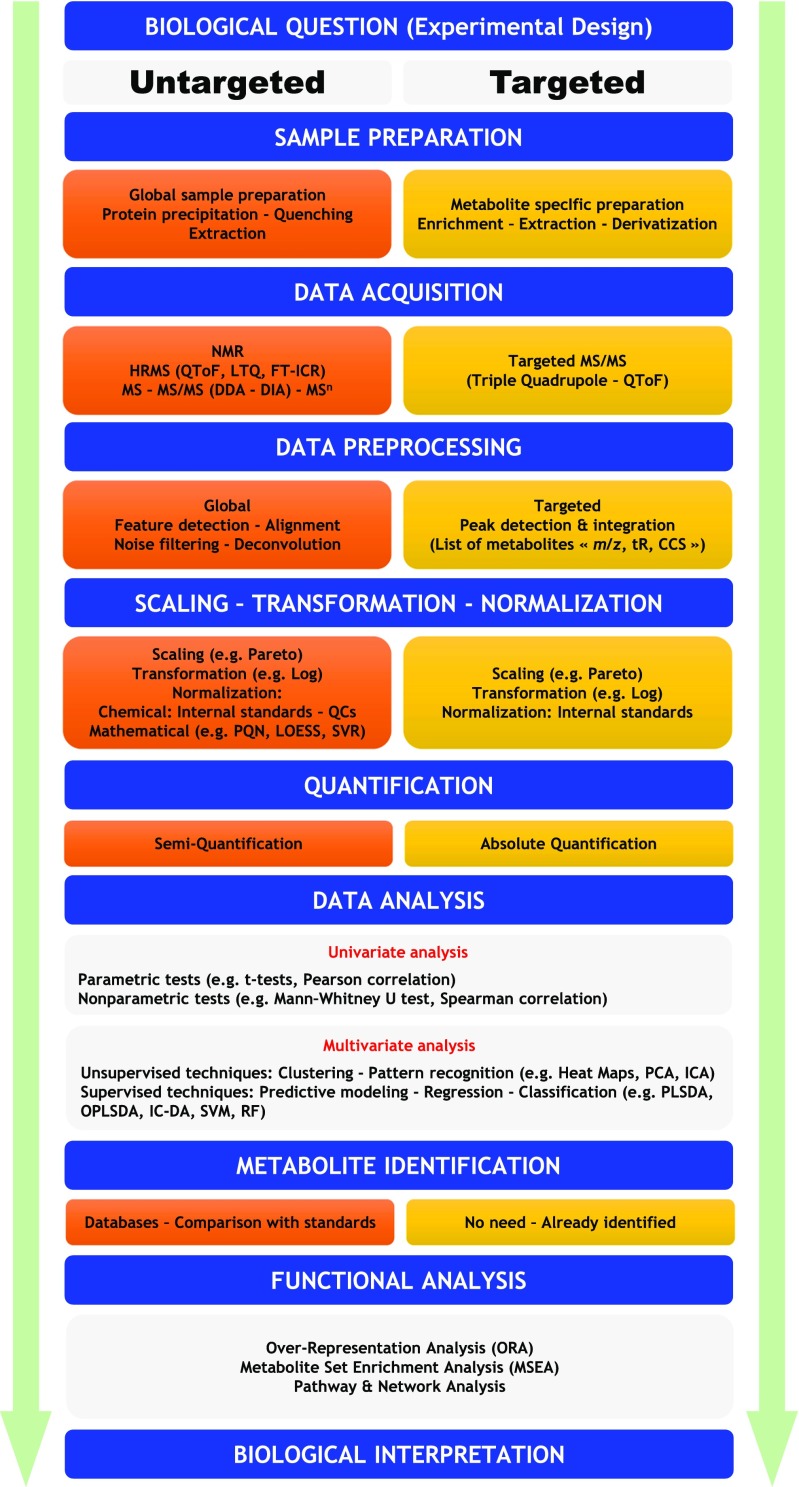
General metabolomics workflow. Metabolomics is divided into two main strategies. A targeted metabolomics is a quantitative analysis or a semiquantitative analysis of a set of metabolites that might be linked to common chemical classes or a selected metabolic pathway. An untargeted metabolomics approach is primarily based on the qualitative or semiquantitative analysis of the largest possible number of metabolites from diverse chemical and biological classes contained in a biological sample. The generated data undergo data analysis step (univariate and multivariate) and functional analysis to get actionable biological insight

A few highly reliable metabolites could be sufficient to a certain extent for diagnostic or monitoring purposes. However, the use of more metabolites for a broader overview is more appropriate for assessing, for example, a biochemical pathway. Thus, metabolomics is obviously an interesting tool to support answering biological questions, especially in biomarker discovery. By definition, a metabolic signature contains a set of disrupted metabolites rather than just a single metabolite, which is plausible because of the relevance of affected metabolic pathways and the network theory underpinning biological systems (Ravasz et al 2002; Bekri 2016). Thus, analytical technologies need to be reliable and robust for high-throughput routine analyses (Zampieri et al 2017). Furthermore, metabolites have qualitatively and quantitatively heterogenic characteristics. Therefore, no single methodology can separate, detect, and quantify the whole metabolome. Thus, multiple analytical techniques and sample preparation strategies are necessary to recover most of the metabolome (Dunn et al 2011a, 2011b). Metabolomics workflows are based on whether the investigator knows a priori what kind of metabolites to assess. A targeted metabolomics approach is defined as a quantitative analysis (absolute concentrations are determined) or a semiquantitative analysis (relative intensities are determined) of a set of metabolites that might be linked to common chemical classes or a selected metabolic pathway. An untargeted metabolomics approach is primarily based on the qualitative or semiquantitative analysis of the largest possible number of metabolites from diverse chemical and biological classes contained in a biological sample. The metabolomics workflow (Fig. 1) comprises the comparative sequential steps of both targeted and untargeted metabolomics analyses. Typical metabolomics experiments aim to analyze as many metabolites as possible in a biological specimen. Several established analytical platforms can enable the semiquantitative assessment (relative intensities) of metabolites. However, the field of metabolomics is increasingly embracing the absolute quantitation of metabolites. The acquired data are extensive and need to be processed and mined to extract insightful biological interpretations. Hence, multivariate data analyses are routinely used to extract information from large metabolomics data sets (Alonso et al 2015). The data can be used to build hypotheses or to explain observations. The identified metabolites associated with an observation provide a holistic overview about the interrogated biological system. The metabolomics workflow generally includes biological problem formulation and experimental design, sample preparation, data acquisition, data preprocessing, data pretreatment, data analysis, network and pathway analysis, and finally biological interpretation (cf. Fig. 1).

### Experimental design

The design of a metabolomics experiment requires consideration of various aspects including sample type, number of samples, replication, data analysis strategy, cost, and time, along with the allocation of human and technical resources. The decisions will lead to answers to different questions. Therefore, a well-defined strategy regarding the tools that will be used for data analysis and interpretation is fundamental, and it should set objective questions and recover appropriate answers. Experimental design and data analysis are tightly related, and the first step in any metabolomics workflow is the clear formulation of the biological problem to be addressed. Depending on the biological problem, the investigator must define the metabolomics approach (targeted vs. untargeted), biological samples (biofluids, tissues, cells, and/or intact organisms), sample size, pooling, experimental conditions (i.e., observational studies, exploratory studies, time series), sampling conditions (frequency of sample collection, quenching to stop enzymatic activity, storage), analytical platforms, and sample preparation protocols. It should be noted that most metabolomics studies are intrinsically comparative; therefore, a group of control samples (samples that did not undergo the investigated condition) and test samples (samples exposed to the investigated condition) are rigorously defined in the experimental design with clear inclusion and exclusion criteria (Broadhurst and Kell 2006). Indeed, subjects are heterogeneous with respect to demographic and lifestyle factors, and this factor is especially important in defining healthy controls. Sample randomization along with sample analysis order and instrument conditions are means to reduce the correlation of confounders. Moreover, advanced statistical experimental design strategies can be used to handle these issues (Dunn et al 2012; Jonsson et al 2015; Boccard and Rudaz 2016).

### Biological samples

Sample collection (time and type), storage, and handling have important impacts on the retrieved metabolic profile (Prentice et al 2013; Burton et al 2014; Yin et al 2015). Thus, these factors have to be standardized to avoid spurious biomarker discovery interpretation (Dunn et al 2011a, 2011b; Emwas et al 2013). Therefore, careful consideration of sampling conditions and handling is needed to provide a reliable metabolic snapshot of the sample at the time it is collected. The primary objective should be to ensure qualitatively and quantitatively consistent and representative samples prior to their collection. Samples can be separated into two general classes: (i) metabolically active samples (intracellular metabolome) and (ii) metabolically inactive samples (extracellular metabolome) (Chetwynd et al 2017). The sample type is chosen based on the biological question being investigated. However, in some studies, the preferred sample type cannot be collected, and a surrogate sample type, such as urine or blood (serum or plasma), has to be used. Compared with intact or extracted tissues, urine and serum or plasma are the most commonly studied biofluids in clinical practice because they are easily obtained and prepared. However, other specialized fluids can be used, including cerebrospinal fluid (Wuolikainen et al 2009; Graham et al 2013), saliva (Dame et al 2015; Kawasaki et al 2015; Mikkonen et al 2015), sweat (Mena-Bravo and Luque de Castro 2014), and even breath (Bach et al 2015; Pijls et al 2016). Dried blood (and other biofluid) spots have also been investigated (Michopoulos et al 2011; Wilson 2011; Prentice et al 2013; Koulman et al 2014) and offer an interesting alternative to conventional liquid samples for generating metabolite profiles. Given their very practical advantages, including low volume, low cost, and handling convenience, dried blood spots are drawing interest as a sampling option for metabolic profiling (Denes et al 2012; Koulman et al 2014; Oliveira et al 2014; Wagner et al 2014). However, DBS exhibit specific challenges due to some disadvantages mainly regarding stability (Adam et al 2011; Michopoulos et al 2011). Several studies showed that DBS storage at a temperature higher than −20 °C leads to metabolite degradation (Fingerhut et al 2009; Prentice et al 2013). Hematocrit and blood volume effects may significantly impact quantitative results (Timmerman et al 2013).

Of note, most metabolomics studies, particularly in clinical metabolomics, include data from a single biofluid, most often blood or urine. However, the biochemical signature in a biofluid denotes complex interactions with different organs, which adds to the interpretative complexity of metabolomics data. This complexity can only be understood by investigating pathophysiological states from a metabolic network perspective, taking into account the local metabolome and its contribution to the systemic metabolome. Different data-driven approaches have been described for handling these issues by using metabolomics data modeling (Do et al 2015; Torell et al 2015).

### Sample preparation

For untargeted metabolomics, minimal sample preparation is generally recommended to avoid metabolite loss. Tissues are often homogenized using manual techniques such as a mortar and pestle or ball grinding with silica particles or stainless steel. The homogenization process is often performed with an extraction solvent, which leads to cell lysis and extraction of the metabolites. Monophasic (water/methanol, water/acetonitrile) or biphasic (water and methanol often along with a nonpolar solvent such as chloroform, dichloromethane, or methyl tert-butyl ether) solvent can be used in extraction systems depending on the planned analysis (Sitnikov et al 2016). The choice of solvent systems depends on whether polar or nonpolar molecules are to be investigated. For cell metabolomics, a quenching step is required before extraction to minimize metabolite modifications. Extraction from cells must be performed as quickly as possible to avoid enzymatic reactions and to improve reproducibility (Ser et al 2015). Sample preparation in cell lines face different challenges. These include growth medium formulation variability and influence of number of passages (Dettmer et al 2011; Bi et al 2013; Martano et al 2015). Using stable isotope dilution and normalization methods may handle recovery issues to achieve reproducible data. For blood samples, the effects of different anticoagulants on plasma profiles are evident as ion suppression or enhancement on metabolite intensity. This affects mainly polar metabolites in EDTA and citrate plasma (Barri and Dragsted 2013). Heparin has been recommended as the anticoagulant in MS-based metabolomics. Other important issues regarding sample handling should be dealt with such as extraction pH, storage time, temperature (Vuckovic 2012; Siegel et al 2014).

The preparation and analysis may be costly in terms of time, which may be quite limiting in the clinical environment. The ability to collect data without sample preparation combined with real-time data analysis would allow rapid clinical decision-making, which would place metabolomics at a higher clinically actionable level (i.e., surgery, pathology). For example, the intelligent knife (iKnife) represents a significant advance in in vivo sampling and real-time metabolomics technology. This process vaporizes tissue and the resultant smoke is transferred into a mass spectrometer to provide real-time clinical decision-making in the operating room (Balog et al 2013).

### Analytical platforms

The analysis of the metabolome raises different challenges compared with other omics analyses, which are based on profiling large molecules built with a simple and limited set of subunits, such as nucleotides for genomics and transcriptomics and amino acids for proteomics. For identification and functional analysis of DNA, RNAs, and proteins, subunit order is what matters; it represents the observed biological complexity. Hence, analytical strategies based on sequencing essentially rely on the incremental detection of the subunits (Athersuch 2016). However, a sequencing concept cannot be applied to metabolites in complex biofluids because the analytical challenge does not lie in cracking any order code; there is no order. The metabolome requires a more complex analytical strategy that allows individual and selective differentiation of metabolites across a wide qualitative and quantitative chemical space. The physicochemical heterogeneity of metabolites adds another layer of complexity to metabolomics studies. In an early scientific paper in the field, Pauling and colleagues described a method using gas chromatographic separation with flame ionization detection to analyze the breath (Pauling et al 1971). Impressive analytical developments have occurred since then. The metabolic profiling technologies that are mainly used now include NMR spectroscopy and MS, sometimes in combination with a gas phase or liquid phase separation method (Alonso et al 2015). These technologies retrieve global, unbiased, and comprehensive chemical information from complex mixtures. For information translation, the resultant high-dimensional spectral data are typically analyzed using chemometric techniques to identify informative metabolic combinations that can be used for either global biomarker discovery or sample classification.

#### Nuclear magnetic resonance spectroscopy

NMR spectroscopy is rapid and nondestructive, and it has the advantage of being highly reproducible and robust. It is based on the absorption and re-emission of energy by the atom nuclei due to variations in an external magnetic field. Different types of metabolomics data can be generated depending on the targeted atom nuclei. However, in the analysis of biological samples, hydrogen is the most commonly used type of nuclei (^1^H–NMR) because of its naturally high abundance in these samples. Other nuclei, such as carbon-13 (^13^C–NMR) and phosphorus-31 (^31^P NMR), can also be used to provide additional information on specific metabolite types. ^31^P NMR is useful for studies of cellular energy states in vivo and ex vivo, but a limitation is the overlapping of ^31^P signals from phosphorylated compounds. NMR spectroscopy is a powerful technology that offers atom-centered information that is crucial for elucidating molecular structures (Emwas et al 2013). The resulting spectral data allow quantification and identification of the metabolites. Peak areas are used for quantification, whereas the spectral patterns permit metabolite identification. The spectral data generated by NMR techniques can be divided into two NMR strategies regarding the frequency axis used. Frequency axes are referenced by the chemical shift expressed in parts per million (ppm). The chemical shift is calculated as the difference between the metabolite resonance frequency and that of a reference substance (Nagana Gowda and Raftery 2017). One-dimensional NMR (1D–NMR) spectra are based on a single frequency axis, where the peaks of each molecule occur within the resonant frequencies of that axis. This method is the most used in high-throughput metabolomics. Two-dimensional NMR (2D–NMR), which is based on two frequency axes, can be used to complement 1D–NMR. Signals are either binned and then analyzed or fitted to patterns of signals corresponding to the metabolites expected to be present in the mixture. ^13^C NMR signals are better resolved, but they exhibit low sensitivity due to a low natural abundance of ^13^C (Markley et al 2017). In 2D–NMR, the second dimension allows separation of overlapping spectral peaks and therefore provides additional and orthogonal chemical information on the investigated metabolites within the analyzed matrix (Larive et al 2015). 2D–NMR methods include ^1^H-^1^H COZY (correlated spectroscopy), ^1^H–^1^H TOCSY (total correlation spectroscopy), and ^1^H–^13^C HSQC (heteronuclear single-quantum correlation) (Emwas et al 2013). Of note, nuclei with low natural abundance, including ^2^H (deuteron), ^13^C, and ^15^N, may serve as excellent metabolic tracers (Fan et al 2016). Despite its relatively low sensitivity, often at the μM level, NMR spectroscopy offers many advantages because it allows rigorous quantification of highly abundant metabolites present in biological fluids, cell extracts, and tissues with minimal or no sample preparation (Fan and Lane 2016). NMR spectroscopy is useful for molecules that are difficult to ionize or require derivatization for MS analysis. NMR spectroscopy also allows the identification of isomeric molecules, and it is the gold standard for determining structures of unknown compounds. Using stable isotope labels, NMR spectroscopy can be used for dynamic assessment of compartmentalization of metabolic pathways, such as metabolite transformations and drug metabolism. Finally, intact tissue NMR imaging and spectroscopy are very appealing for in vivo metabolic investigations (Verma et al 2016). The main drawback of NMR methods is its low sensitivity and resolution compared with MS-based methods (Emwas 2015).

#### Mass spectrometry

Mass spectrometry is an analytical technique that retrieves chemical data from the gas-phase ions produced from a sample. The ions generate different peak patterns that define the fingerprint of the original molecule in the form of a mass-to-charge ratio (*m/z*) and a relative intensity of the measured chemical features (e.g., metabolites). The sample is introduced into the mass spectrometer via the sample inlet, an ion source generates gas-phase ions, a mass analyzer separates the ions according to their *m/z*, and a detector generates an electric current from the incident ions that is proportional to their abundances (Murray et al 2013). A sample can be directly injected into a mass spectrometer such as in direct infusion mass spectrometry (DIMS) (González-Domínguez et al 2017). The major drawback is the ion suppression effect, which leads to metabolite information loss and prohibits separation of isomers. Mass analyzers can be used alone or in combination with the same type of mass analyzer or with different mass analyzers (hybrid instruments). Such combinations are the foundation for the analytical mode of tandem mass spectrometry (MS/MS). In MS/MS, the ions that arrive at the first mass analyzer (precursor ions) are selected, then fragmented in a collision cell. The fragmented ions are separated according to their *m/z* in a second mass analyzer and then detected. Different operation modes are possible, including data dependent analysis (DDA) and data independent analysis (DIA). In DDA, a fixed number of precursor ions whose *m/z* values were recorded in a survey scan are selected using predetermined rules and are subjected to a second stage of mass selection in an MS/MS analysis (Mann et al 2001). Modes include single reaction monitoring (SRM) or multiple reaction monitoring (MRM), which is the application of SRM with parallel detection of all transitions in a single analysis. In DIA, all precursor ions within a defined *m/z* window undergo fragmentation. The analysis is repeated as the high resolution mass spectrometer progresses through the full selected *m/z* range (Plumb et al 2006). This process yields accurate metabolite quantification without being limited to profiling predefined metabolites of interest (Zhou et al 2017). One caveat of applying this to metabolomics, is that in a complex sample it may exhibit co-eluting compounds with similar fragments. Therefore, MS/MS acquisition on a wider range of masses may lead to specificity issues related to fragment ions from multiple parent ions. To handle this, the MS^E^ technique alternates between “high energy” and “low energy” scans on a Q-TOF instrument. MS^E^ has a fast duty cycle (~0.3 s) which makes the technique compatible with ultra-high performance liquid chromatography (UHPLC). Furthermore, the use of ion mobility separation prior to metabolite fragmentation may improve precursor selectivity. For some mass analyzers, such as quadrupole ion traps, several steps of MS/MS can be performed. For example, the fragmented ions can be further fragmented and detected. The experiment is called multiple-stage mass spectrometry (MS^n^, n refers to the number of MS steps). MS/MS and MS^n^ improve structural identification, combining information from both molecular and fragmented ions generated from precursor ions. The main performance characteristics of mass analyzers are (1) mass accuracy, or mass resolving power, which is related to the ability of an MS analyzer to generate distinct signals for two ions with a small *m/z* difference; (2) mass range, which is the range of *m/z* over which a mass spectrometer can detect ions to record a mass spectrum; (3) sensitivity; (4) scan speed; and (5) duty cycle time, which is the fraction of ions that effectively reach the detector in the mass spectrometer. The mass analyzer choice is mainly based on the type of metabolomics approach to be carried out, targeted or untargeted. Single quadrupole (Q), triple quadrupole (QqQ), quadrupole ion trap (QIT), and Orbitrap (OT) are suitable for targeted metabolomics because of their sensitivity and duty cycle characteristics. In comparison, dynamic range, mass accuracy, and resolution power are the main characteristics of a mass analyzer to be used in untargeted metabolomics studies. Time of flight (TOF), quadrupole time of flight (QTOF), Fourier transform ion cyclotron resonance (FTICR), and OT are the most used mass analyzers for this purpose. The principle underlying TOF and QToF involves the time required for ions to travel a flight tube. Ions are accelerated in an electric field, reaching a linear velocity that depends on their *m/z* ratio. The velocity can reach 10,000 per second scan speed, with a mass error of 5 ppm. A QTOF mass analyzer is a hybrid instrument that can generate high-resolution MS/MS spectra (Forcisi et al 2013). FTICR is an ultra-high-resolution (10^5^–10^7^ depending on the detection time and magnetic field) mass analyzer that uses cyclotron frequency in a fixed magnetic field to measure *m/z* ions at the cost of relatively slow acquisition rates (typically 1 Hz). In the same way, the OT is also an FTMS instrument, which is based on harmonic ion oscillations in an electrostatic field. Ions are trapped around a central electrode, and ion oscillation frequencies are used to measure the *m/z* values. The OT provides high mass resolution (>100,000 FWHM), high mass accuracy (2–5 ppm), and an acceptable dynamic range. However, the scan speed is inversely related to mass resolution. Recently, the high-field Orbitrap has provided a resolution above 1,000,000 at *m/z* 300–400 with 3 s detection time, using an absorption mode (Denisov et al 2012). A wide range of instrumental and technical variants are currently available for MS spectrometry. These variants are mainly characterized by different ionization and mass selection methods (Glish and Vachet 2003).

Because of the matrix effect limit and potential isomers, MS is generally preceded by a separation step in metabolomics. This step reduces the complexity of a biological sample and allows sequential MS analysis of the different molecules. Different separation methods coupled to MS have been described, such as liquid chromatography (LC-MS) (Want et al 2010; Want et al 2013), gas chromatography (GC-MS) (Chan et al 2011), and capillary electrophoresis (CE-MS) (Ramautar et al 2015). Thus, metabolites with different chemical properties will spend different amounts of time (retention time, t_R_) in the separation dimension. These different separation methods enhance the sensitivity and the dynamic range of MS and provide complementary and orthogonal molecular information.

- Liquid chromatography

LC-MS is widely used in metabolomics because of its analytical versatility, covering separation performance of different classes of molecules, from very polar to very lipophilic compounds. This high versatility is achieved through the variety of chromatographic columns along with stationary phases (Kuehnbaum and Britz-McKibbin 2013). The LC separation basics depend on physico-chemical properties, such as hydrophobicity, molecular size, and polarity. The separation of compounds occurs in a chromatographic column composed of a stationary phase with polar or lipophilic properties. When polar stationary phase columns are used, the method is referred to as normal-phase liquid chromatography (NPLC); when nonpolar stationary phase columns are used, the method is called reversed-phase liquid chromatography (RPLC). The choice of LC columns depends on the polarity of the metabolites and the analytical scope. To analyze nonpolar and/or weakly polar metabolites, nonpolar C18 and C8 columns are mostly used for untargeted metabolomics (Forcisi et al 2013). However, for hydrophilic, ionic, and polar compounds, hydrophilic interaction liquid chromatography (HILIC) is recommended. HILIC is similar to NPLC, but it differs because of the mobile phase, which is composed of a polar and/or aprotic organic solvent miscible in water that is easier to use with electrospray-mass spectrometry (Tang et al 2014). Recently, Prinsen et al reported a HILIC tandem MS-based method for the analysis of 36 underivatized plasma aminoacids in an 18 min run (Prinsen et al 2016). Sowell et al developed a HILIC tandem MS-based method for the quantification of free and total carnitine avoiding the derivatization step (Sowell et al 2011). For further details on HILIC-based metabolomic strategies, the reader may refer to a recent comprehensive review (Tang et al 2014). Multiple-column strategies could be used for more extensive metabolome coverage (Haggarty and Burgess 2017). Recently, RPLC and HILIC columns with a smaller internal diameter (e.g., 1 mm) and shorter length have drawn interest in metabolomics. These columns allow the use of regular LC flow rates with very high back pressure. Thus, instruments that can operate at very high pressure—ultra-performance liquid chromatography (UHPLC)—coupled to mass spectrometry have been introduced to improve metabolite coverage and detection. UHPLC methods allow increased resolution, better sensitivity, and lower ion suppression. As a result, better metabolome coverage is obtained in comparison with conventional HPLC. Moreover, lower solvent consumption is observed because of the low flow rate (150–250 μL/min) (Kaufmann 2014). It is to be noted that chromatographic conditions are crucial in regarding metabolome coverage and the unbiased proprieties of untargeted metabolomics studies (Boudah et al 2014).

- Gas chromatography

GC-MS is often used for analysis of volatile compounds and molecules with low vapor pressure, such as lipids, long-chain alcohols, amides, alkaloids, sugar alcohols, and organic acids. In addition, using derivative techniques widens the coverage of GC-MS. GC-MS has been accepted as a robust metabolomics platform because of its selective separation, reproducibility, and robustness. The greatest advantage of GC-MS is that its ionization mode is highly reproducible and standardized (based on electron ionization at 70 eV) across GC-MS systems worldwide and across different vendors (Kopka et al 2005), which has allowed comprehensive GC-MS mass spectral libraries such as NIST and FiehnLab to be established (Vinaixa et al 2016). As a result, GC-MS has been a set and reliable platform for MS-based metabolomics. The main limitation of GC-MS is the necessary derivatization step for some metabolite classes. In metabolomics, derivatization usually uses oximation and a silylation/chloroformate reagent. This step is time consuming, hampers the throughput, and can introduce error by adding analytical variability (Moros et al 2017). Moreover, GC-MS metabolome coverage is limited by the stationary phase stability as well as the thermal stability of metabolites and their derivatives (Kaal and Janssen 2008).

- Capillary electrophoresis

Capillary electrophoresis (CE) offers an orthogonal separation mechanism. CE-specific characteristics, such as high efficiency and resolution, high throughput, and, importantly, the ability to assess the most polar compounds without derivatization, have made CE an attractive method for metabolomics (García et al 2016). CE-MS was the last pre-ionization separation technique to be paired with MS in metabolomics. Capillary zone electrophoresis (CZE) is the simplest and most commonly used CE mode because of its principle of separation and its broad application to the analysis of diverse samples, spanning small to large biomolecules. In CZE, analytes are separated according to their intrinsic differential electrophoretic mobility in a capillary filled with separation buffer under the influence of an electric field. The mobilities depend on the ion *m/z* and the viscosity of the medium (García et al 2016). The main drawback of CZE is that neutral molecules are not separated. To overcome this disadvantage, other CE modes have been developed, such as micellar electrokinetic chromatography, capillary isotachophoresis, capillary isoelectric focusing based on pH gradient, capillary electrochromatography, capillary gel electrophoresis, and affinity capillary electrophoresis. Because of its simplicity, CZE is the preferred CE mode in metabolomics. Recently, DiBattista et al described an elegant high throughput multiplexed separation platform based on CE-MS combined with temporal signal pattern recognition for screening of different inherited metabolic diseases (IMD). Their result showed comparable performances with flow injection analysis. Furthermore, the authors described new biomarkers for galactosemia screening N-galactated amino acids (DiBattista et al 2017). Despite the recent technical advances of CE-MS, its use in metabolomics is still limited compared with NMR spectroscopy and chromatography-based methods. For more details about CE-MS applications in metabolomics, the reader may refer to a recent review (Rodrigues et al 2017).

- Ion mobility and multidimensional strategies

Another gas phase separation, ion mobility spectrometry (IMS), (Hill et al 1990), is drawing interest in metabolomics (Dwivedi et al 2010; Wickramasekara et al 2013; Paglia et al 2014; Smolinska et al 2014; Hauschild et al 2015; Maldini et al 2015; Paglia et al 2015). In general, the multidimensional coupling of different separation techniques requires that the resolution obtained from each anterior separation must be largely preserved as the analytes pass to the following dimensions. This preservation is particularly difficult when all analytes travel along the same path during the analysis, as is the case for tempo-dispersive techniques. Thus, the solution is to incrementally increase the sampling frequency of each subsequent time dimension so that multiple measurements are obtained within a fixed time interval. In this way, the arrival time in each anterior dimension can be reassembled based on the integrated signal of subsequent dimensions. This strategy is commonly used when coupling condensed phase separations such as GC, LC, or CE to MS. IMS is an appealing post-ionization separation method that is based on molecular size, shape, and charge. It is typically performed on a millisecond timescale, which can be perfectly nested between chromatography (seconds) and high-resolution MS detection (microseconds) timescales. Hence, coupling IMS with high-resolution mass spectrometry and chromatography (LC-IMS-MS) provides additional analytic selectivity without significantly compromising the speed of MS-based measurements. As a result, the MS dimension affords accurate mass information, while the IMS dimension provides molecular, structural, and conformational information through the determination of the ion collision cross-section (CCS), which is a valuable and predictable chemical descriptor. Indeed, ion mobility spectrometry adds a separation dimension to the hybrid MS instruments, allowing a higher analytical coverage of complex biological mixtures (Fenn and McLean 2008; Fenn et al 2009; Kliman et al 2011; Paglia et al 2014; Tebani et al 2016a, b). One important feature of IMS is its ability to separate isomers (Domalain et al 2013); the predictability of the CCS and peak width for one isomer mainly depend on ion diffusion (Jeanne Dit Fouque et al 2015; Harper et al 2016; Zhou et al 2016). Furthermore, exploring a multivectorial space containing retention time, accurate mass, and CCS obtained by the combination of multiple separation methods with MS allows valuable measurement integration, which enhances molecular identification and consequently biomarker discovery (May et al 2015; Sherrod and McLean 2015).

- Toward real-time MS-based metabolomics

Recent introduction of ambient ionization sources has significantly increased the high throughput of global metabolic profiling analysis. These techniques permit direct sampling of complex matrices under ambient conditions, and they include atmospheric solids analysis probe (Twohig et al 2010), desorption electrospray ionization (Eberlin et al 2013; Ferreira et al 2015; Kerian et al 2015), and rapid evaporative ionization MS methods (Balog et al 2013; Balog et al 2015). These techniques can provide real-time, interpretable MS data on biofluids and tissues, in vivo and ex vivo, and they are reshaping high-throughput real-time metabolome analysis in different areas (Arentz et al 2017; Dunham et al 2017). For example, in many surgeries, visually distinguishing between healthy and diseased tissues is often difficult. It requires time-consuming biopsies and immuno-staining procedures to be performed by experienced trained histopathologists during surgery. By eliminating this need for external tissue histotyping, techniques such as the iKnife could open the way to true real-time precision surgery. For more details about the use of ambient MS in clinical diagnosis, refer to a recent and detailed review by Ifa and Eberlin (Ifa and Eberlin 2016). Table 1 presents a comparison between different analytical strategies used in metabolomics.

**Table 1:** Comparison of main analytical technologies in metabolomics

Platform	Technique	Identification dimensions	Principle	Advantages	Limits
Nuclear magnetic resonance	1 Dimension 2 Dimensions	Chemical shift Chemical shift × chemical shift	Uses interaction of spin active nuclei (^1^H, ^13^C, ^31^P) with electromagnetic fields, yielding structural, chemical, and molecular environment information	Nondestructive Highly reproducible Exact quantification possible Minimal sample preparation Molecular dynamic and compartmental information using diffusional methods Relatively high throughput Availability of databases for identification	High instrumentation cost Overlap of metabolites Low sensitivity
Mass spectrometry	Direct injection (DI-MS)	*m*/*z*	Uses a nanospray source directly coupled to MS detector. It does not require chromatographic separation.	Very high throughput High sensitivity No cross-sample contamination No column carryover Low-cost analysis Automated analysis Low sample volume requirement Allows MS imaging	Samples not recoverable (destructive) No retention time information, which gives limited specificity Inability to separate isomers Subjected to significant ion suppression phenomenon High ionization discrimination (ESI)
	Liquid chromatography (LC-MS)	Time × *m*/*z*	Uses chromatographic columns that enables liquid phase chromatographic separation of molecules followed by MS detection (suitable for polar to hydrophobic compounds)	Minimal sample preparation (protein precipitation or dilution of biological sample) High-throughput capability UHPLC can be coupled to any type of MS Flexibility in column chemistry widening the range of detectable compounds High sensitivity	Samples not recoverable (destructive) Very polar molecules need specific chromatographic conditions Retention times are highly dependent on exact chromatographic conditions Batch analysis Lack of large metabolite databases High ionization discrimination (ESI)
	Gas chromatography (GC-MS)	Time × *m/z*	Uses chromatographic columns that enables gas phase chromatographic separation of molecules followed by MS detection (suited for apolar and volatile compounds)	Structure information obtained through in-source fragmentation Availability of universal databases for identification High sensitivity Reproducible	Samples not recoverable (destructive) Requires more extensive sample preparation Only volatile compounds are detected Polar compounds need derivatization Low ionization discrimination
	Capillary electrophoresis (CE-MS)	Time × *m*/*z*	Uses electrokinetic separation of polar molecules paired with a mass spectrometry detector	Excellent for polar analysis in aqueous samples Measures inorganic and organic anions Low running costs	Samples not recoverable (destructive) Relatively low throughput profiling
	Ion mobility spectrometry (IMS-MS)	Time × *m*/*z* (CCS × *m*/*z)*	Uses a uniform or periodic electric field and a buffer gas to separate ions based on charge, size, and shape paired with mass spectrometry	Very robust and reproducible (ability to determine collision cross-section, which is a robust chemical descriptor) High peak capacity High selectivity Separation of isomeric and isobaric compounds Very high throughput	Samples not recoverable (destructive) CCS and mass are highly correlated parameters, which limits the orthogonality of the method

## Quality control management

Quality control (QC) is defined as the set of procedures that a laboratory performs during or immediately after the analysis to demonstrate the quality of the data. Validation of an analytical strategy is key in QC strategies. Method validation procedures are used to confirm that a given analytical procedure is suitable for its intended use and give an overview about the method and the produced data quality (Kadian et al 2016). In targeted metabolomics, data integrity and analytical quality assessment is tightly regulated and different guidelines have been issued by regulatory bodies regarding bioanalytical method validation criteria (Kadian et al 2016). These criteria may include accuracy, precision, specificity, limit of detection, limit of quantitation, and linearity. Furthermore, external QC schemes are well established for interlaboratory assessment such ERNDIM scheme used by biochemical genetics laboratories (Fowler et al 2008). However, regarding untargeted metabolomics, no regulatory guidelines have been published. Hence, the metabolomics community is willing to propose QC methods to assess untargeted metabolomics data and enhance data integrity (Dunn et al 2017). The QC rational is often purpose-driven. Since, the purpose of untargeted metabolomics is to find statistically significant metabolites retrieved from as many as possible of detected chemical features in a given biological sample through differential analysis. Hence, this purpose will determine the validation parameters since absolute quantification is not the primary objective in untargeted metabolomics. Comparisons are considered valid provided all the samples are assessed under the same conditions using a precise analytical method. The signal change should be related to the abundance of the chemical features and should be as independent as possible from other instrumental and analytical variations. Different metrics are used to clean, assess, and filter the initial data using QC samples. In metabolomics, it is often recommended to analyze QC samples at regular intervals across an entire run in order to monitor the experimental data (Dunn et al 2012). The QC samples are prepared by pooling the study samples to represent all included samples. Using QC, it is possible to assess each feature in the data regarding its presence in the QCs. The percentage detection rate defines how consistently the feature is detected across the samples. Thus, features that are not present in a defined minimum number of QC can be filtered out from the data. Often, 50% cut-off is applied (Dunn et al 2012). Response drifts can also be monitored using QC throughout the data acquisition. As data acquisition often takes a long time, it is common to observe response drifts, which lead to intra- and inter-batch variation. Several methods are available to remove intensity drifts using feature intensities of the QC along with the experimental run order (Shen et al 2016). Correction factors are used to remove intensity drifts in each feature by dividing the intensity by the correction factor. Repeatability is another quality criterion than can be assessed using QC. To have a good repeatability and be retained in final dataset, each feature in the QCs should exhibit low relative standard deviation (RSD) across all the QC samples. RSD for each feature is calculated by dividing the sample standard deviation by the sample mean. Features with high RSD values should be cleaned from the data. Thresholds have been suggested ranging from 20% to 30% (Dunn et al 2012). However, this may be flexible depending on the experimental design. Finally, a series of QC samples with varying dilutions can be prepared and analyzed within the experimental run, could be used to assess features quality. The dilution factors can be regressed against the corresponding intensities of each feature in the data. Features intensity must be correlated to the matrix concentration of the diluted QC samples in order to be retained for further analysis. The features with low correlation coefficient (R^2^) are thus removed. Thresholds ranging from 50% and 70% are often suggested (Lewis et al 2016). However, inspecting the distribution of the R^2^ values may help in setting the threshold. This filter being independent of the study design is, therefore, applicable in both small and large-scale studies. Recently, the dilution strategy has been elegantly illustrated by DiBattista et al in a CE-MS based method with integrated QC strategy that allows inter-sample comparisons and inter-batch signal drift (DiBattista et al 2017).

## Applications in inherited metabolic diseases

With the multi-metabolite quantitative abilities of metabolomics, the future of IMD diagnosis may be found in the developing area of metabolomics. Targeted MS-based metabolomics is already widely used and implemented in IMD newborn screening national programs worldwide (Therrell et al 2015). Several IMD are routinely screened using targeted MS-based metabolomics methods such as organic acidurias, aminoacidopathies, and fatty acid oxidation disorders (Pitt et al 2002; Pitt 2009; Pitt 2010; Spacil et al 2013; Auray-Blais et al 2014). However, combining the already existing tools with actionable data analysis strategies, metabolomics is very appealing for better and effective diagnosis. For example, an integrated strategy for IMD screening, using both targeted and untargeted approaches, have been recently proposed by Miller et al. The method provides actionable diagnostic information for IMD. The authors have successfully diagnosed 21 IMD disorders using plasma metabolite measurements through metabolomics (Miller et al 2015). For more details on metabolomics potential in IMD, the reader may refer to recent comprehensive reviews (Piras et al 2016; Tebani et al 2016a, b).

## Conclusion

Metabolomics is intrinsically a multidisciplinary field that requires different analytical, biological, and bioinformatics skills. Substantial advances have occurred in analytical chemistry for metabolomics strategies for better chemical data extraction from biological samples. These advances have had a substantial impact on metabolomics workflows by simplifying analytical protocols and introducing more robust systems. However, to go a step further to translate metabolomics into an actionable exploratory and ultimately a diagnostic tool, issues that need to be addressed include streamlining and automating sample preparation, improving analytical throughput by using faster separation (or no separation, if using DIMS), and introducing orthogonal analytical dimensions such as IMS-MS in metabolomics. NMR and chromatography-based platforms are still the well-established technologies for metabolomics studies. LC-MS and GC-MS are the most adopted analytical platforms in clinical metabolomics. Still, for a more comprehensive metabolome coverage, implementation of multiplatform approaches is necessary. To reach next-generation metabolomics, further advances are urgently needed in analytical strategies for reliable identification and absolute quantification. Finally, standardization regarding sample handling and analytical procedures is a big issue for larger clinical studies and wide adoption of metabolomics, particularly, in clinical environments. The choice of the technology to implement depends mainly on the scope of the laboratory, the financial constraints, and the preexisting resources and expertise.
